# Cannabinoids in the management of behavioral, psychological, and motor symptoms of neurocognitive disorders: a mixed studies systematic review

**DOI:** 10.1186/s42238-022-00119-y

**Published:** 2022-03-14

**Authors:** Anees Bahji, Natasha Breward, Whitney Duff, Nafisa Absher, Scott B. Patten, Jane Alcorn, Darrell D. Mousseau

**Affiliations:** 1 Department of Psychiatry, University of Calgary, 2500 University Drive NW, Calgary, Alberta T2N 1N4 Canada; 2 Cannabinoid Research Initiative of Saskatchewan (CRIS), Saskatchewan, Canada; 3 College of Pharmacy and Nutrition, University of Saskatchewan, Saskatoon, Saskatchewan Canada; 4 Department of Community Health Sciences, University of Calgary, Calgary, Alberta Canada; 5 Cell Signalling Laboratory, Department of Psychiatry, College of Medicine, University of Saskatchewan, 107 Wiggins Road, Saskatoon, Saskatchewan S7N 5E5 Canada

## Abstract

**Aim:**

We undertook this systematic review to determine the efficacy and safety of cannabis-based medicine as a treatment for behavioral, psychological, and motor symptoms associated with neurocognitive disorders.

**Methods:**

We conducted a PRISMA-guided systematic review to identify studies using cannabis-based medicine to treat behavioral, psychological, and motor symptoms among individuals with Alzheimer's disease (AD) dementia, Parkinson’s disease (PD), and Huntington’s disease (HD). We considered English-language articles providing original data on three or more participants, regardless of design.

**Findings:**

We identified 25 studies spanning 1991 to 2021 comprised of 14 controlled trials, 5 pilot studies, 5 observational studies, and 1 case series. In most cases, the cannabinoids tested were dronabinol, whole cannabis, and cannabidiol, and the diagnoses included AD (*n* = 11), PD (*n* = 11), and HD (*n* = 3). Primary outcomes were motor symptoms (e.g., dyskinesia), sleep disturbance, cognition, balance, body weight, and the occurrence of treatment-emergent adverse events.

**Conclusions:**

A narrative summary of the findings from the limited number of studies in the area highlights an apparent association between cannabidiol-based products and relief from motor symptoms in HD and PD and an apparent association between synthetic cannabinoids and relief from behavioral and psychological symptoms of dementia across AD, PD, and HD. These preliminary conclusions could guide using plant-based *versus* synthetic cannabinoids as safe, alternative treatments for managing neuropsychiatric symptoms in neurocognitive vulnerable patient populations.

## Introduction

In the general population, the risk of Alzheimer’s disease (AD) is 1% at 60 years of age and doubles every 5 years afterwards (Alzheimer Society of Canada 2010). The National Population Health Study of Neurological Conditions estimates that AD accounts for annual health care system and caregiver costs totalling $10.4 billion, with an expected increase of 60% by 2031 (Public Health Agency of Canada 2014). Generally, home-care and long-term care are the largest contributors to direct costs; additionally, family caregivers contribute significant costs (19.2 million unpaid hours of care in 2011, a number projected to double by 2031).

Behavioral and psychological symptoms of dementia (BPSD) are considered the most common complications of any type of dementia, e.g., as high as 90% in most types of dementia and more than 95% in AD (Ikeda et al. 2004; Cerejeira et al. 2012). BPSD can exacerbate cognitive decline and physical dysfunction in this patient group (Mintzer et al. 1998), and one of the most common Neuropsychiatric Symptoms (NPS) associated with BPSD in AD is anxiety (Benoit et al. 1999). Other symptoms include agitation, aggression, depression, apathy, delusions, and hallucinations, as well as changes in sleep and appetite (Cerejeira et al. 2012).

Despite the frequency and severity of BPSD, there are no clear pharmacotherapeutic options. The several medications used off-label have modest efficacy and significant associated risks, emphasizing an unmet clinical need for BPSD (Ballard and Waite 2006). Some authors suggest that the most common BPSD in AD is anxiety, present in more than 65% of BPSD cases (Benoit et al. 1999), which has led to the suggestion that anxiety (rather than depression, another risk factor for AD) might be a better predictor of cognitive decline (Bierman et al. 2009). The pharmacologic treatment of BPSD, including anxiety, is often inferred from studies in younger cohorts of individuals with anxiety but lacking a dementia diagnosis (Baldwin et al. 2005). Treatment options for mood and anxiety disorders in the elderly often include antidepressants (e.g., selective serotonin reuptake inhibitors (SSRIs), serotonin-noradrenaline reuptake inhibitors), and benzodiazepines (Linden et al. 2004). Current treatments for BPSD include SSRIs, atypical antipsychotics, second-generation antipsychotics, non-tricyclic antidepressants, and short-acting benzodiazepines (Tampi et al. 2016), but treatment responses to these medications are varied, and the pharmaceutical choice depends more so on the presence and severity of adverse events (AEs) rather than on the effectiveness of a chosen drug. AEs can include increased risk of hip fractures/falls, accelerated cognitive decline, and death from cerebrovascular events (Reus et al. 2016; Vigen et al. 2011; Tampi et al. 2016). The Institute for Safe Medication Practices (ISMP) maintains a Beers List outlining those drugs to avoid in the older adult due to an increased risk for harm (American Geriatrics Society 2015). The list includes benzodiazepines, tricyclic antidepressants, and antipsychotics. Furthermore, haloperidol and risperidone—two of the most widely prescribed antipsychotics for BPSD (De Deyn et al. 1999; Suh et al. 2006)—have been shown to activate apoptotic events in mammalian cell cultures and exacerbate cell death induced by the AD-related β-amyloid peptide (Wei et al. 2006).

Dementia is challenging to treat due to the breadth of associated symptoms and often requires complex polypharmacy with complicated AE profiles. The search for a therapeutic alternative to control BPSD in AD patients has recently turned to isolates from the *Cannabis sativa* plant, e.g., cannabinoids (Liu et al. 2015), some of which show promise as anxiolytics (Fusar-Poli et al. 2009) and in the management of depression and bipolar disorder (Ashton et al. 2005). The related literature is ambiguous, but there is also a suggestion that cannabinoids might relieve depression secondary to a life-limiting illness, such as HIV, cancers, multiple sclerosis, or hepatitis C (Brunt et al. 2014). However, the lack of evidence-based information on the safety, tolerability, and general effectiveness of cannabinoids has promoted reluctance amongst physicians to authorize cannabis or related extracts to manage BPSD.

Cannabinoids exert their effects by interacting with the endocannabinoid system (ECS), particularly cannabinoid 1 (CB1R) and cannabinoid 2 (CB2R) receptors. CB1Rs are abundantly located throughout the body with prominent expression in the central nervous system, while CB2Rs are located more peripherally in immune cells and tissues (Lu and Mackie 2020). The ECS is a vital neuromodulatory system associated with several psychiatric, neurodegenerative, and motor disorders such as schizophrenia, anorexia, AD, Parkinson’s disease (PD), and Huntington disease (HD) (Fernandez-Ruiz et al. 2015; Basavarajappa et al. 2017).

Results from preclinical and clinical studies have suggested that the administration of cannabis is associated with improvements in BPSD (including agitation and sleep disturbances) and weight and pain management in AD patients (Sherman et al. 2018). Although cannabis is associated with an increased risk of euphoria, drowsiness, and psychosis, previous trials with AD patients have shown that AEs are generally well tolerated at the doses administered (Sherman et al. 2018). Therefore, attention is shifting to cannabinoids such as cannabidiol (CBD), which exerts beneficial effects on the brain without eliciting the ‘high’ associated with its better-known and more widely studied counterpart Δ^9^-tetrahydrocannabinol (THC). As the population ages, improving quality of life and independence is becoming increasingly essential. Thus, a better understanding of how cannabinoids may benefit the dementia patient is critical, not only to those directly involved but ultimately to our increasingly burdened health care system. To this end, we chose to undertake an evidence-based systematic review to examine the efficacy and safety of CBM as a potential treatment option for BPSD. The review centers on AD and included PD and HD as these two neurocognitive disorders also have a significant BPSD component to their clinical presentation (Cloak and Al Khalili 2021; Gelderblom et al. 2017).

## Methods

### Protocol and registration

There is no pre-registered protocol. However, we followed the Preferred Reporting Items for Systematic Reviews and Meta-analyses (Liberati et al. 2009).

### Eligibility criteria

We followed the population-intervention-comparison-outcome-study design framework to define eligibility. We restricted eligibility to studies involving adults receiving treatment for AD/dementia, PD, or HD and/or its associated symptoms. Eligible interventions included any CBM, including whole cannabis or synthetic cannabinoids. Eligible outcomes included any BPSD-related measure, such as improvement in symptom severity. Eligible study designs were full-text articles supplying data on three or more participants. We excluded non-English studies due to a lack of available translation resources. We also excluded studies with concurrent administration of prescribed pharmacotherapeutics in addition to the cannabinoids—as this may have confounded evaluation of the primary intervention. Because of the limited number of studies that met the broad inclusion criteria, we opted to keep case studies and surveys even though these most often did not include a placebo condition. However, we acknowledge that these types of studies usually do not inform questions of therapeutic efficacy or effectiveness.

### Information sources and search

With the support of a research librarian at the University of Saskatchewan, we searched MEDLINE, International Pharmaceutical Abstracts, and EMBASE from inception to March 2021 (Appendix 1). We also reviewed the Food and Drug Administration (FDA) clinical trial registry in August 2021 for all studies about BPSD as well as reference lists of systematic review articles and other relevant articles to supplement the electronic search.

### Study selection

Reviewers (NB, NA, and AB) screened records electronically using Mendeley to remove duplicates. Next, another two reviewers (NB and WD) screened unique records by title/abstract for relevance to the review. After obtaining the full-text copies of articles relevant to the topic, reviewers (NB, WD, and AB) screened the remaining records for review inclusion. Finally, two external co-authors (JA and DM) settled discrepancies across the study selection stages.

### Data collection process and data items

The following data items were collected using piloted forms: author, year, study location, number of patients enrolled in the study (“n”), study type/design, the primary endpoint, dementia type/severity, type of product used (CBD, THC, both), route of administration, dose, dose regime, comparator, study length, primary endpoint results, AEs, number of patients that withdrew from the study (with reasons, if reported), and notes of interest (comorbidities, author affiliations). Data extracted also included the study’s primary outcome and conclusions. The first reported outcome was interpreted as the primary outcome in the absence of a specified primary outcome and no power calculation.

### Risk of bias in individual studies

The reviewers independently assessed ‘study quality’ using the Downs and Black (Downs and Black 1998) quality assessment (Appendix 2) with a slight modification concerning the scoring of item 27 of the assessment that refers to the power of the study. According to an available range of study powers, item 27 is rated on whether the report includes a power calculation or not as suggested for use in systematic methodological reviews (MacLehose et al. 2000).

### Summary measures

Although we had planned to conduct a quantitative meta-analysis before reviewing the literature, we were unable to do so given the heterogeneity of the identified studies. Instead, we supplied a narrative summary of the findings.

## Results

### Study selection

**Fig. 1:**
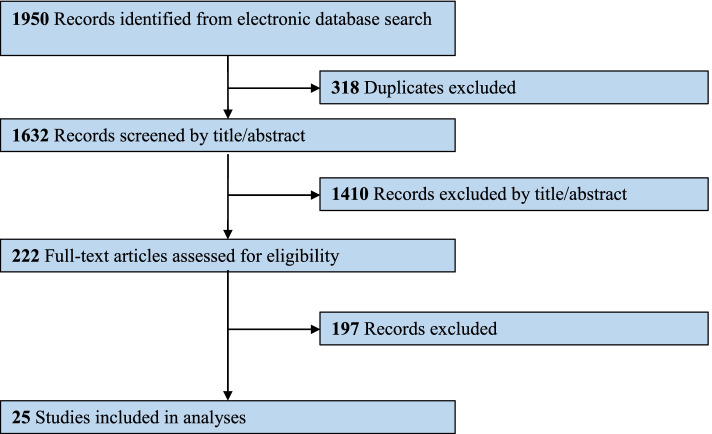
Diagram of literature review

Of the initial 1950 articles identified, 222 remained potentially eligible after removing duplicates and screening remaining abstracts. Ultimately, 25 studies (Ahmed et al. 2015; Balash et al. 2017; Bruce et al. 2018; Carroll et al. 2004; Chagas et al. 2014a; Chagas et al. 2014b; Consroe et al. 1991; Curtis et al. 2009; Herrmann et al. 2019; Lopez-Sendon Moreno et al. 2016; Lotan et al. 2014; Mahlberg and Walther 2007; Mesnage et al. 2004; Shelef et al. 2016; Shohet et al. 2017; Sieradzan et al. 2001; van den Elsen et al. 2015a; van den Elsen et al. 2015b; van den Elsen et al. 2017; Venderova et al. 2004; Volicer et al. 1997; Walther et al. 2006; Walther et al. 2011; Woodward et al. 2014; Zuardi et al. 2009) met inclusion criteria for the review (Fig. 1).

### Study characteristics

The final review included articles published from 1991 to 2021 (Table 1). The majority (*n* = 15) were randomized, controlled trials, and there was one retrospective cohort study. The remaining nine studies included open-label pilot studies (*n* = 5), surveys (*n* = 3), and a case series (*n* = 1). We included the latter nine studies in our narrative summary, even though these types of studies do not often inform therapeutic efficacy or effectiveness questions. The most commonly evaluated cannabinoids were dronabinol (*n* = 10), whole cannabis (*n* = 5), cannabidiol (*n* = 4), nabilone (*n* = 3), nabiximols (*n* = 2), and cannabinoid receptor antagonists (SR 141716, SR 48692, SR 142801) (*n* = 1). The studies included patients with AD/dementia (*n* = 11), PD (*n* = 11), and HD (*n* = 3).

**Table 1:** Study characteristics (*n* = 25)

Study	Design	Sample	Intervention(s)	Findings	Quality
Ahmed et al. 2015	12-week crossover RCT (*n* = 10)	Adults with dementia with significant neuropsychiatric symptoms	Dronabinol; 1.5–3 mg vs. placebo, p.o.	98 mild AEs were reported during the study period. Thirteen reported AEs were possibly related to study drugs (dronabinol or placebo). No SAEs related to THC were reported.	22
Balash et al. 2017	Retrospective cohort study (*n* = 47)	Adults with PD	MC; 0.2–2.25 g/day, mostly smoked (84%)	MC improved PD symptoms in the majority (82.2%), while two (4.4%) reported no difference and six (13.3%) reported a worsening of symptoms. 59.6% reported AEs: confusion (17%), anxiety (17%), hallucinations (17%), short-term amnesia (6.5%), psychosis (2.1%), cough (34.9%), dyspnea (4.7%), dizziness (12.8%), and unsteadiness (15.6%).	12
Bruce et al. 2018	Retrospective cohort study (*n* = 30)	Patients receiving medicinal cannabis for a qualifying health condition	MC; 60% smoked cannabis flowers	MC was most frequently (60% of participants) reported as an alternative to prescription medications. Minor AEs were reported with MC compared to prescription medications.	10
Carroll et al. 2004	10-week crossover RCT (*n* = 19, ages 18–78)	19 PD patients with levodopa-induced motor symptoms (dyskinesia)	THC; 0.25 mg/kg and 0.125 mg/kg CBD vs placebo, p.o.	UPDRS dyskinesia scores worsened (*p* = 0.09), and mild AEs were reported in both groups. All AEs improved by dose reduction, and there were no SAEs. 37 mild AEs reported total: 18 physical, with dry mouth most common (*n* = 4), and 20 psychological, with drowsy/lethargic most common (*n* = 9).	20
Chagas et al. 2014a	6-week RCT (*n* = 21)	Adults with idiopathic PD with motor symptoms (dyskinesia)	CBD; capsules; 75–300 mg (*n* = 14) vs. placebo (*n* = 7)	No difference (*p* = 0.544) in mean UPDRS score variations between the three treatment groups. No AEs were observed in any of the groups through UKU or verbal reports. No difference (*p* = 0.855) between groups in BDNF levels, measured complementary to subjective AE reports.	20
Chagas et al. 2014b	6-week case series (*n* = 4)	Patients with PD in RCT (Chagas et al. 2014a) that also fulfilled criteria of: a) complete clinical assessment for RBD and b) at least two episodes of complex sleep-related behaviors per week.	CBD; capsules 75 mg/day (*n* = 3), 300 mg/day (*n* = 1)	Prompt, substantial, and persistent reduction in the frequency of RBD-related events in all four cases. AEs were not reported.	9
Consroe et al. 1991	15-week crossover RCT (*n* = 18)	Adults with HD not taking antipsychotics with motor symptoms (chorea)	CBD; 10 mg/kg vs. placebo, p.o., b.i.d.	Treatment response favored CBD with a lower median M and Q chorea severity score (11.5) than placebo (13.7, *p* = 0.71). No difference between CBD and placebo for cannabis side-effect inventory. Three male patients withdraw after completing 5, 6, and 10 weeks of the study for reasons unrelated to the trial.	20
Curtis et al. 2009	15-week crossover RCT (*n* = 44)	Adults with HD with motor symptoms (chorea)	Nabilone; 1–2 mg vs. placebo, p.o., b.i.d.	No difference in UHDRS total motor score between treatment groups. There were three SAEs, seven withdrawals from the study (two due to SAEs).	22
Herrmann et al. 2019	14-week RCT (*n* = 39)	Adults with dementia and NPS	Nabilone; 1–2 mg vs. placebo, p.o.	Nabilone reduced agitation. However, it increased the risk of sedation and worsened cognition.	27
Lopez-Sendon Moreno et al. 2016	12-week crossover RCT (*n* = 25)	Adults with HD	Dronabinol; 2.7 mg THC/2.5 mg CBD per spray, 12 sprays per day	No differences in motor, cognitive, or functional outcomes against placebo, or in symptomatic effects.	24
Lotan et al. 2014	1-day prospective cohort study (*n* = 22)	Adults with PD	MC; smoked 0.5 g per day for 2 months	UPDRS total motor score improved (*p* < 0.001) from baseline (33.1 ± 13.8) to 30 min after (23.2 ± 10.5) *cannabis* consumption. One patient had hypoglycemia that resolved after oral glucose intake, and one patient complained of dizziness. AEs included sleepiness, palpitations, and bad taste.	17
Mahlberg and Walther 2007	2-week RCT (*n* = 24)	Adults with AD and NPS	Dronabinol; 2.5 mg vs. melatonin 3 mg, p.o.	The nocturnal activity was significantly reduced (*p* = 0.001) in the dronabinol group. No AEs were observed.	13
Mesnage et al. 2004	9-day RCT (*n* = 25)	Adults with PD and motor fluctuations and levodopa-induced dyskinesia	SR 141716; 20 mg; SR 48692 180 mg; SR 142801; 200 mg vs. placebo, p.o.	No significant differences in the delay before turning “on” between groups. No AEs were observed. One patient did not complete the study due to unexpected nausea (SR 48692), symptoms disappeared within 24 h.	14
Shelef et al. 2016	4-week RCT (*n* = 11)	Adults with AD and BPSD	Dronabinol; 5–15 mg vs. placebo, p.o., b.i.d.	MMSE showed modest trend (*p* = 0.08) of change from baseline (10.3) to 4 weeks (11.0). There were three AEs reported, including confusion at 10.0 mg dose, a fall and resulting pelvic fracture, and dysphagia, who withdrew from the study.	16
Shohet et al. 2017	40-week prospective cohort study (*n* = 20)	Adults with PD	Cannabis; smoked (*n* = 18) or vaporized (*n* = 2)	Decrease (*p* < 0.0001) in UPDRS motor function score from before (38.1 ± 18) to 30 min after (30.4 ± 15.6) cannabis consumption. No AEs were observed.	11
Sieradzan et al. 2001	2-week crossover RCT (*n* = 9)	Adults with PD and stable levodopa-induced dyskinesia	Nabilone; 0.03 mg/kg vs. placebo, p.o.	Nabilone reduced (*p* < 0.05) median total dyskinesia score (17, range 11 to 25) over placebo (22, range 16 to 26). All patients experienced a postural fall in systolic blood pressure, but no difference between groups. AEs included sedation, dizziness, hyperacusis, partial disorientation, and visual hallucinations. Two patients withdrew after nabilone from vertigo and symptomatic postural hypotension.	19
van den Elsen et al. 2015a	3-week RCT (*n* = 50)	Adults with AD, vascular, or mixed dement and clinically relevant NPS (NPI ≥ 10)	Dronabinol; 4.5 mg (*n* = 24) vs. placebo (*n* = 26), p.o., t.i.d.	NPI was reduced in both treatment groups after 14 (*p* = 0.002) and 21 days (*p* = 0.003). There was no difference (NPI change score = 3.2 [− 3.6 to 10.0]) between groups at 21 days. There was no difference in AEs between groups (16 vs. 14). Three patients withdrew: pneumonia, nausea, and withdrew consent.	27
van den Elsen et al. 2015b	14-week crossover RCT (*n* = 22)	Adults with AD, vascular, or mixed dement and clinically relevant NPS (NPI ≥ 10)	Dronabinol; 1.5–3 mg (*n* = 22) vs. placebo (*n* = 22), p.o., b.i.d.	No difference in effect on NPI between dronabinol and placebo. There were 184 AEs, distributed between dronabinol (94) and placebo (93) treatments. Four SAEs occurred in three patients, all requiring a prolongation of hospitalization. None of the SAEs were judged to be related to the study drug. Two patients withdrew, one due to the occurrence of malignancy and extensive use of psychotropic rescue medication.	25
van den Elsen et al. 2017	8-week crossover RCT (*n* = 18)	Adults with AD, vascular, or mixed dementia and clinically relevant NPS (NPI ≥ 10)	Dronabinol; 1.5 mg (*n* = 18) vs. placebo (*n* = 18), p.o., b.i.d.	Static balance as assessed by body sway (roll angle) was similar with eyes opened (*p* = 0.10), but significantly higher (0.32 ± 0.6°/s, *p* = 0.05) after dronabinol versus placebo administration.	24
Venderova et al. 2004	Retrospective cohort study (*n* = 630)	Adults with PD	Whole cannabis; smoked	45.9% (*n* = 39) described mild or substantial alleviation of PD symptoms in general. There were no AEs reported.	8
Volicer et al. 1997	12-week crossover RCT (*n* = 15)	Adults with AD and food refusal	Dronabinol; 2.5 mg vs. placebo, p.o., b.i.d.	Bodyweight increased (*p* = 0.006) over the 12-week study period regardless of the order of treatment. However, the treatment effect was more significant (*p* < 0.017) when participants received dronabinol during the first (7.0 ± 1.5 lbs) versus second (2.3 ± 1.7 lbs) period, compared to placebo during the first (4.6 ± 1.3 lbs) versus second (1.7 ± 2.3 lbs). One patient withdrew from a seizure and two from infections, and one died of a heart attack.	18
Walther et al. 2006	2-week RCT (*n* = 6)	Adults with dementia and nighttime agitation	Dronabinol; 2.5 mg vs. placebo, p.o.	Actigraphy nocturnal motor activity during the last 5-nights of treatment. Dronabinol reduced (*p* = 0.028) nocturnal motor activity from baseline (24.29) to 14 days (3.76). No AEs were observed.	17
Walther et al. 2011	4-week crossover RCT (*n* = 2)	Two patients with AD or mixed dementia with agitation	Dronabinol; 2.5 mg vs. placebo, p.o.	No severe AEs or deterioration occurred during the trial.	16
Woodward et al. 2014	Retrospective cohort study (*n* = 40)	Inpatients with dementia and NPS	Dronabinol; variable dose	Dronabinol decreased agitation and improve global ratings of function, sleep duration, and proportion of meals consumed, but caused mild AE.	20
Zuardi et al. 2009	4-week RCT (*n* = 6)	Adults with PD and treatment-resistant psychosis	CBD; flexible dose, starting at 150 mg vs. placebo, p.o.	Reduced BPRS scores (18.5 to 5.5, *p* < 0.001) in BPRS total scores from baseline (median 18.5) to 4 weeks (5.5) with CBD treatment. No AEs were observed.	17

*Abbreviations***:** *AD* Alzheimer’s disease, *AE* adverse event, *b.i.d*. *bis in die*/twice a day (total dose indicated, divided into two equal doses), *BPSD* behavioral and psychological symptoms of dementia, *BPRS* brief psychiatric rating scale, *CBD* cannabidiol, *CDR* clinical dementia rating, *DSM-IV* Statistical Manual of Mental Disorders, *HD* Huntington’s disease, *MC* medical cannabis, *MMSE* mini-mental state examination, *MoH* Israeli Ministry of Health, *NA* not applicable, *NINDS-AIREN* National Institute of Neurological Disorders and Stroke and the Association Internationale pour la Recherche et l’Enseignement en Neurosciences, *NINCDS-ADRDA* National Institute of Neurological and Communicative Disorders and Stroke and the Alzheimer’s Disease and Related Disorders Association, *NPI* neuropsychiatric inventory, *NPS* neuropsychiatric symptoms, *PD* Parkinson’s disease, *p.o*. *per os*/by mouth, *RBD* REM sleep behavior disorder, *RCT* randomized controlled trial, *SAE* serious adverse event, *THC* Δ9-tetrahydrocannabinol, *t.i.d. ter in die*/three times a day (total dose indicated, divided into three equal doses), *UHDRS* unified Huntington’s disease rating scale, *UKPDSBB* UK Parkinson’s disease society brain bank, *UKU* Udvalg for kliniske undersogelser, *UPDRS* Unified PD Rating Scale

### Risk of bias within studies

Based on the modified Downs and Black assessment tool (MacLehose et al. 2000), the checklist's maximum score is 28, with 20–28 being ‘good’, 15–19 being ‘fair’, and 14 and below being viewed as ‘poor’. The quality scores indicated articles were of ‘good’ quality (*n* = 12), ‘fair’ quality (*n* = 6), and ‘poor’ quality (*n* =7) (Appendix 3 and 4). Within the ‘good’ to ‘fair’ quality categories, the majority were crossover RCTs (Ahmed et al. 2015; Carroll et al. 2004; Consroe et al. 1991; Curtis et al. 2009; Herrmann et al. 2019; Lopez-Sendon Moreno et al. 2016; Sieradzan et al. 2001; van den Elsen et al. 2015b; Volicer et al. 1997; Walther et al. 2011; van der Hiel et al. 2017), one parallel RCT (van der Leeuw et al. 2015), a retrospective cohort study (Woodward et al. 2014). Although such studies usually do not inform therapeutic efficacy or effectiveness questions, we identified several ‘good’ to ‘fair’ quality open-label pilot studies (Lotan et al. 2014; Shelef et al. 2016) and a ‘good’ quality case series (Chagas et al. 2014b). Within the ‘poor’ quality category, two were parallel RCTs (Chagas et al. 2014a; Mahlberg and Walther 2007), one was a crossover RCT (Mesnage et al. 2004), and the other four included surveys (Balash et al. 2017; Bruce et al. 2018; Venderova et al. 2004) and an open-label pilot study (Shohet et al. 2017). Articles did not consistently identify a primary outcome in the introduction or methods, most were underpowered, and there were common methodological issues in more than half of the studies, including several which reported probability values, the lack of sample representativeness of the entire population, and lack of intervention compliance reporting, or measurement bias (if the studies were not blinded, this could be a significant factor in any interpretation).

### Cannabinoids for Parkinson’s disease and Huntington’s disease

For those with PD or HD, the focus of studies was usually on dyskinesia or chorea improvements. Of these, none reported safety as the primary outcome, and only one of the PD studies reported dementia symptoms, measured using the Brief Psychiatric Rating Scale (BPRS), which was initially developed to assess symptom domains in schizophrenia, but has been used in AD/dementia clinical trials (e.g., (Sultzer et al. 2008)). We realize several versions of the BPRS measure the same rating items but can include more items than others. The version was often not specified in our review, yet as all studies based on assessments using the BPRS are within-person studies, we felt this would not affect our interpretations. Other reported primary outcomes included PD symptoms (*n* = 2), dyskinesia (*n* = 2), symptoms of REM sleep behavioral disorder (RBD) (*n* = 1), delay before turning “on” (*n* = 1), and Unified PD Rating Scale (UPDRS) dyskinesia (*n* = 1), motor (*n* = 2), or total (*n* = 1) score. CBM improved non-motor symptoms (including reducing falls, depression, and pain, while promoting sleep) in PD subjects (Balash et al. 2017), while CBM worsened UPDRS scores, although these did not reach significance (Carroll et al. 2004). Another study found no difference in mean UPDRS scores between treatment groups (Chagas et al. 2014a). However, two studies indicated an improvement (decrease) in UPDRS score, including motor (rigidity, tremor) and non-motor (sleep, pain) symptoms, with smoked (whole) cannabis use (Lotan et al. 2014; Shohet et al. 2017). There was a reduction in the frequency of RBD-related events (Chagas et al. 2014a) and a lower median M and Q chorea score with CBD use (Consroe et al. 1991). In contrast, there was no difference in UHDRS total motor score with nabilone, which reduced the total dyskinesia score in subjects (Curtis et al. 2009). Finally, a ‘fair’ quality, open-label study indicated four weeks of CBD improved the BPRS score (improved psychotic symptoms, without any effect on motor symptoms) in six PD patients (Zuardi et al. 2009).

### Cannabinoids for dementia

In general, the studies of individuals with dementia reported BPSD, such as agitation, sleep disturbance, food refusal, and nocturnal motor activity. All dementia studies focused on individuals with AD, though most included individuals with mixed dementia (e.g., vascular or frontotemporal features). Two of these studies reported AEs, and two reported on the Neuropsychiatric Inventory (NPI) as the primary outcome. Other reported primary outcomes included nocturnal activity (*n* = 1), cognition (based on the Mini-Mental State Examination; MMSE) (*n* = 1), static balance (*n* = 1), and body weight (*n* = 1). A few (13%) studies included patients with HD (*n* = 3), with only one reporting a primary outcome of absence of serious adverse events (SAEs; *n* = 1) and the other two reporting primary outcomes of the M and Q chorea severity scale (*n* = 1) and total motor score using the Unified Huntington’s Disease Rating Scale (UHDRS), a tool to assess the clinical features and course of HD (*n* = 1). The remaining two studies included patients with dementia and patients with chronic diseases that use medical cannabis. Four weeks of THC decreased the NPI scores in AD patients (e.g., delusions, aggression, apathy, and sleep) (Shelef et al. 2016), while another study found that THC decreased NPI/NPS scores after 14 and 21 days, but scores were no different from placebo after the 21-day mark (van den Elsen et al. 2015a). Another study found no difference between the dronabinol and placebo group on NPI/NPS score (van den Elsen et al. 2015b). Dronabinol increased body weight (improvement in anorexia and behavioral disorders) (Volicer et al. 1997) and reduced nocturnal motor activity from baseline to 14 days (Walther et al. 2006).

### Safety

Five studies utilized CBD products, with no AEs observed in two (Chagas et al. 2014b; Zuardi et al. 2009), mild AEs in one (Carroll et al. 2004), and AEs were not reported in one (Chagas et al. 2014a). The fifth study found abnormal laboratory results in more than 50% of the patients (Consroe et al. 1991). However, these results were limited to 12 of 70 tests ran, and abnormalities were not remarkably outside the normal ranges. Furthermore, these abnormalities did not coincide with subjective reports of cannabis side effects, as there were no differences in inventory when comparing CBD and placebo (Consroe et al. 1991). Based on these results, we could not identify any definitive concerns regarding the safety of CBD-based products for use in dementia. While a large number of mild AEs were reported (98 total), only six were possibly related to dronabinol; two (fatigue, dizziness) at the lower dose of 1.5 mg and four (fatigue, agitation) at the higher dose of 3.0 mg. Further, no significant differences in AEs were reported with dronabinol than placebo in either period of a crossover study (Ahmed et al. 2015). Participants receiving dronabinol reported similar AEs as those receiving placebo, and episodic memory scores decreased similarly between groups (van den Elsen et al. 2015a; van den Elsen et al. 2015b). Although few withdrawals from AEs were reported, one of the two patients who withdrew in one of the trials did so due to extensive psychotropic rescue medication use (van den Elsen et al. 2015b).

### Summary of findings

This systematic review summarized twenty-five articles exploring CBM for the treatment of neurocognitive disorders. We found that CBM formulations containing higher CBD concentrations were associated with improved motor symptoms, such as dyskinesia and chorea, associated with HD and PD. CBM with higher THC concentration also appeared to show an association with reduced severity of BPSD, such as sleep disturbance and agitation. Overall, CBM appeared to be well tolerated, as the occurrence of treatment-emergent AEs was low; however, CBM with higher THC content could worsen baseline cognition. These preliminary conclusions could guide using plant-based versus synthetic cannabinoids as safe, alternative treatments for managing neuropsychiatric symptoms in neurocognitive vulnerable patient populations.

## Discussion

### Summary of evidence

This review of the literature has revealed the complexity associated with cannabinoid-based treatments in elderly populations. While some studies report a lack of effect of THC on neuropsychiatric symptoms (van den Elsen et al. 2015a), others have shown improvement in BPSD with the use of synthetic THC, e.g., dronabinol [the (-) enantiomer of THC] or nabilone [a racemic mix of THC] (Liu et al. 2015; Shelef et al. 2016; Woodward et al. 2014). A recent systematic review targeting safety and efficacy found THC treatments resulted in more AEs than placebo or prochlorperazine in older participants, with side effects ranging from more common ones such as sedation and drowsiness to less frequent but more severe ones, such as cardiac arrhythmia and grand mal seizures (van den Elsen et al. 2014). Elsewhere, CBD was shown to be anxiolytic (Fusar-Poli et al. 2009), a property of this compound that is so remarkable that it even attenuates the anxiety often associated with THC use (Zuardi et al. 1982; Crippa et al. 2011). CBM has also been shown to reduce the use of other prescription medicines (Abuhasira et al. 2018). In general, the lack of evidence-based information on the safety, tolerability, and general effectiveness of CBM leads to a reluctance among physicians to authorize CBM for treatment, including a management option for BPSD in AD, PD, and HD. Polypharmacy and more frequent comorbidities introduce additional complexity to novel prescription compounds such as cannabis (Mahvan et al. 2017).

The present review included 25 studies and encompassed a broad range of cannabinoids, including whole cannabis, THC, cannabidiol, pharmaceutical THC (e.g., dronabinol, nabilone), and cannabis receptor antagonists. Unfortunately, the range of outcomes, including dyskinesia and chorea severity, and a broad range of BPSD, precluded meaningful meta-analyses. However, considering the balance of risks and benefits, there appears to be more consistent evidence for the use of CBD in treating the motor symptoms of HD and PD. In contrast, our systematic review does identify several ‘good’ and ‘fair’ (and one ‘low’) quality studies based on pharmaceutical cannabinoids, such as nabilone and dronabinol, that suggest effectiveness in relief from agitation in the context of dementia across AD, PD, and HD.

It is not clear why this distinction between plant-extracted and pharmaceutical THC (and related compounds) may exist. One possibility is the influence of the ‘entourage effect’ in the plant-extracted preparations, reflecting any one of 150 cannabinoids or terpenes and secondary metabolites, any one of which might be biologically active (Ferber et al. 2020). Indeed, their potential interactions with other receptor families including the vanilloid receptor (TRVP1) (Bisogno et al. 2001) (implicated in pain pathways; (Caterina and Julius 2001)) and monoaminergic receptors, such as the 5-HT1A and 5-HT2 receptors, the β-adrenergic and α-adrenergic receptors, and dopamine receptors (Bisogno et al. 2001; Seeman 2016; Marchese et al. 2003), could contribute to outcomes in measures of BPSD and motor phenotypes. Interestingly, THC does not appear to exert any effect on dopamine D2 receptors (Marchese et al. 2003), explaining why the purer forms of THC, e.g., nabilone and dronabinol, were less likely to be associated with improvement in motor deficits in the current systematic review. However, one cannot discount other interactions with molecules as diverse as the peroxisome proliferator-activated receptor (transcription factor involved in glucose and lipid homeostasis as well as inflammation), fatty acid amide hydrolase and monoacylglycerol lipase (two enzymes that degrade endogenous cannabinoid ligands), and COX-2 (mediates production of prostaglandins) (Di Marzo and Piscitelli 2015).

Surprisingly, very few studies have reported potential side effects and AEs associated with applying CBM to treating adults with neurocognitive disorders—a crucial limitation from a medication development perspective. However, a previous meta-analysis of nine randomized controlled trials of different CBM as adjunctive treatments for BPSD due to AD found preliminary evidence for their efficacy and tolerability (Bahji et al. 2020a). Furthermore, across those nine trials, there were few reported AEs. Regardless, the review concluded that CBM should not be viewed as first-line therapy. Their use is typically limited to treatment-resistant cases due to poor study quality and the theoretical risk of worsening cognition—particularly when there is polypharmacy. The current review should improve clinical decision-making as it includes a broader search—encompassing non-dementia cognitive disorders, such as HD and PD—that has highlighted a critical distinction between plant-extracted and synthetic cannabinoids, and their potential in relief from motor symptoms (in HD and PD) and management of BPSD (across AD, PD, and HD), respectively.

## Limitations and future research directions

Although the modified Downs and Black Checklist (MacLehose et al. 2000) is appropriate for the quality assessment of randomized and non-randomized trials, we applied this same assessment tool to other types of articles (e.g., observational), ultimately assigning lower scores to several non-RCT articles. Some studies were potential duplications, such as the 2017 report by van den Elsen and colleagues (van den Elsen et al. 2017), which appeared to have been a secondary analysis of a 2015 study by the same group (van den Elsen et al. 2015b). AEs tended to be frequent and mild, but usually not the study’s primary outcome and may have been incompletely reported. Furthermore, considerable heterogeneity existed that included product variety (e.g., route of administration, formulations, doses), different intervention lengths, and multiple scales/methods to assess the efficacy or effectiveness of CBM, making it difficult to compare studies and outcomes. Blinding in studies with CBM is a challenge, as subjects can often tell if they are on an active drug or placebo due to side effects. Few studies attempted to blind the participants or blind both participants and physicians to the treatment option.

This review included both observational and RCTs. Several studies lacked power calculation. Other review limitations included focusing on English language studies and a lack of contact information for study authors for further follow-up. Consequently, we based all conclusions solely on the articles’ information, and there was a theoretical risk of publication bias. We acknowledge that our quality assessment tool may have had different thresholds of ‘good’, ‘fair’, and ‘poor’ quality studies compared with other tools and could lead to some subjectivity when deciding how studies may be pooled. We also acknowledge that combining good, fair, and poor quality studies can lead to a false sense of precision around the overall validity of our conclusions. Still, any bias was likely mitigated by combining independent reviewers and additional unbiased reviewers to resolve discrepancies.

We completed a search of the FDA clinical trial registry, which includes NIDA’s clinical trial database, for all studies about BPSD, and identified 63 ongoing/completed trials. However, none of the recorded studies involved a CBM, underpinning the critical need for considering CBMs in human trials to address this knowledge gap.

Equally striking was the lack of consideration of sex/gender in most studies, which precluded any possibility of a generalizable conclusion regarding sex/gender influences within this systematic review. However, the inclusion of sex as a nominal variable in any cannabinoid-related clinical research, particularly in the context of BPSD, should be a high priority given that sex hormones might exert an influence on response to cannabinoids (for example, THC-mediated relief of pain being dependent on the estrous cycle (Wakley and Craft 2011) and the regulation of cannabinoid receptor binding by estrogens (Riebe et al. 2010)). In contrast, cannabinoids might exert sex-dependent influences on metabolism (more so in males) and mood, e.g., anxiety and depression (more so in females) (Fattore and Fratta 2010). In addition, the higher incidence of AD/dementia in women (Ott et al. 1998) and the higher incidence of PD/dementia in men (Reekes et al. 2020) suggest a need to consider a sex-by-cannabinoid response for any neurodegenerative disorder and warrants additional research in this area.

Finally, as cannabis and CBM may have AEs on cognitive processes, it is essential to know whether potential improvements observed in some reviewed studies are primary or secondary to improvement in other domains (e.g., anxiety and depression). However, this has not been previously explored. There are also no data on accelerated cognitive decline in those with dementia who use cannabis. Cannabis is also associated with dependence and withdrawal syndromes, with one review showing that cannabis withdrawal symptoms affect nearly half of individuals with regular or dependent cannabis use (Bahji et al. 2020b). As dependence and withdrawal phenomenon have not been previously explored among older adults or those with neurocognitive disorders, these are important areas for future research to explore in relation to CBM as a treatment.

## Conclusion

Our systematic review has revealed a paucity of studies in this area. The reports identified herein already suggest an apparent association between CBD-based products and relief from motor symptoms in HD and PD, and an apparent association between synthetic cannabinoids and relief for BPSD (across all three diagnoses). Given the known safety issues with more traditional pharmacotherapeutic management options, this summary of the available evidence can be used to guide the physician on the potential differential benefit of plant-based *versus* synthetic cannabinoids for treating the problems that neuropsychiatric symptoms produce for patients with neurocognitive vulnerability. Before any clinical recommendation can be made, it will be essential to replicate some randomized clinical trials.

## Figures and Tables

**Table 4:**
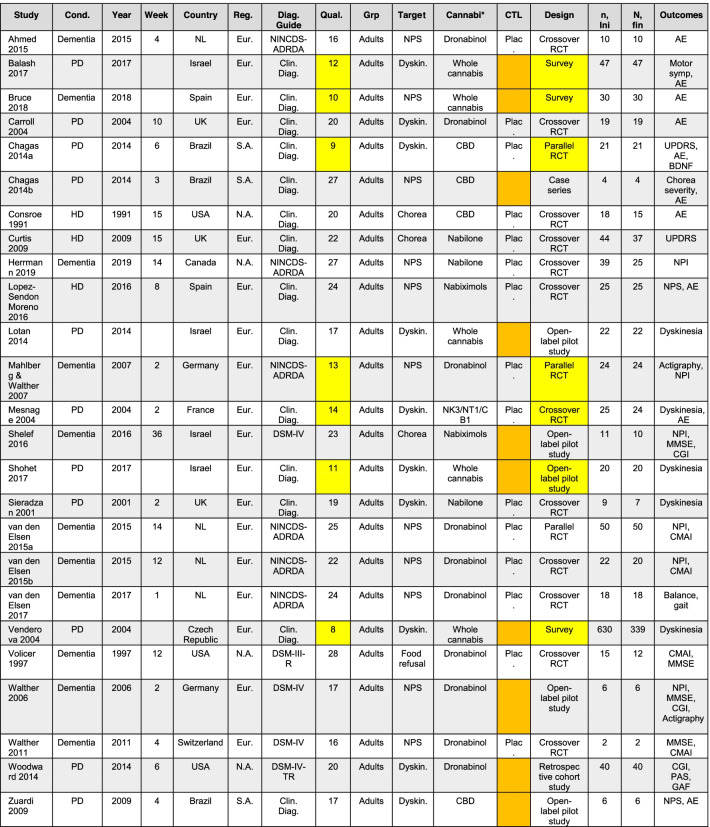
Summary of study parameters

*Abbreviations*: *AD* (dementia) Alzheimer’s disease, *AE* adverse event, *BDNF* brain-derived neurotrophic factor, *CGI* Clinical Global Impression, *Clin. Diag*. clinical diagnosis, *Cond.* condition (diagnosis), *CMAI* Cohen-Mansfield Agitation Inventory, *Diag. Guide* diagnostic guide), *DSM* Diagnostic and Statistical Manual of Mental Disorders (version III or IV or IV-TR/Text Revision), *Dyskin.* dyskinesia, *Eur.* Europe, *GAF* Global Assessment of Functioning, *HD* Huntington’s disease, *MMSE* mini-mental state examination, *N.A.* North America, *NINCDS-ADRDA* National Institute of Neurological and Communicative Disorders and Stroke and the Alzheimer’s Disease and Related Disorders Association, *NL* Netherlands, *NPI* neuropsychiatric inventory, *NPS* neuropsychiatric symptoms, *PD* Parkinson’s disease, *PAS* Pittsburgh Agitation Scale, *Plac* placebo, *Reg.* region, *RCT* randomized controlled trial, *S.A.* South America, *UPDRS* Unified PD Rating Scale, *UK* United Kingdom, *USA* United States of America, *Week* week (treatment duration), *Year* year (of study), Yellow highlights: studies with a ‘poor’ quality rating (< 14 on the Modified Downs and Black Checklist (Appendix 2); Orange highlights: studies lacking a placebo treatment

**Table 5:**
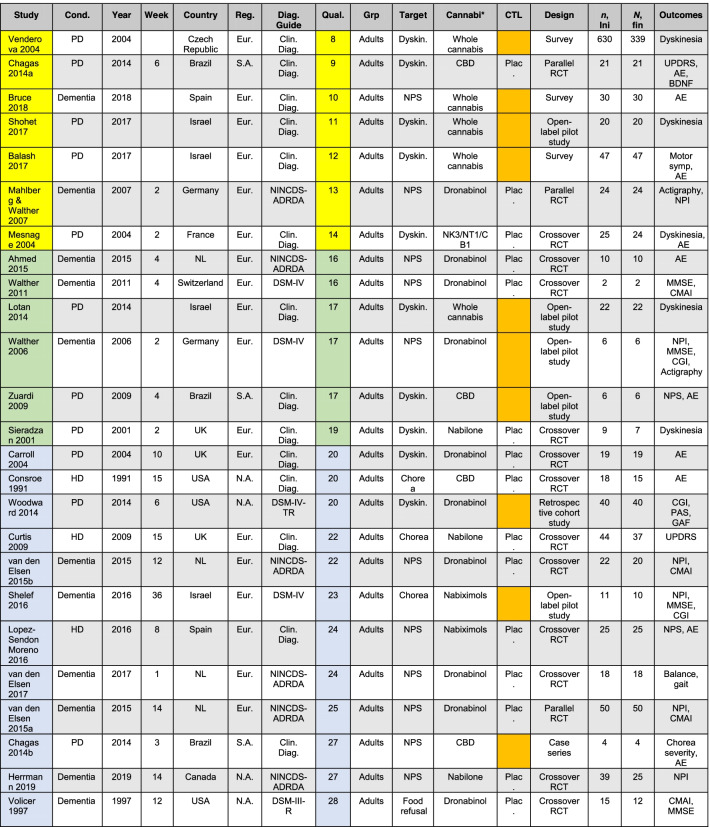
Summary of study parameters (alternative order based on quality ratings)

*Abbreviations*: *AD* (dementia) Alzheimer’s disease, *AE* adverse event, *BDNF* brain-derived neurotrophic factor, *CGI* Clinical Global Impression, *Clin. Diag.* clinical diagnosis, *Cond.* condition (diagnosis), *CMAI* Cohen-Mansfield Agitation Inventory, *Diag. Guide* diagnostic guide, *DSM* Diagnostic and Statistical Manual of Mental Disorders (version III or IV or IV-TR/Text Revision), *Dyskin*. dyskinesia, *Eur.* Europe, *GAF* Global Assessment of Functioning, *HD* Huntington’s disease, *MMSE* mini-mental state examination, *N.A.* North America, *NINCDS-ADRDA* National Institute of Neurological and Communicative Disorders and Stroke and the Alzheimer’s Disease and Related Disorders Association, *NL* Netherlands, *NPI* neuropsychiatric inventory, *NPS* neuropsychiatric symptoms, *PD* Parkinson’s disease, *PAS* Pittsburgh Agitation Scale, *Plac* placebo, *Reg.* region, *RCT* randomized controlled trial, *S.A.* South America, *UPDRS* Unified PD Rating Scale, *UK* United Kingdom, *USA* United States of America, *Week* week (treatment duration), *Year* year (of study); Studies are rated as ‘poor’ quality (< 14; yellow highlight), ‘fair’ quality (16-19; green highlight), and ‘good’ quality (20–28; blue highlights), with all quality rating based on the Modified Downs and Black Checklist (Appendix 2); Orange highlights: studies lacking a placebo treatment

## Appendix 1

Table 2

**Table 2:** Search strategy: MeSH terms are bolded

**Embase (Embase Classic + Embase 1947 to 2017 week 41)**	
**Dementia-related terms**	**Cannabis-related terms**
Dementia Dementias Alzheimer disease Alzheimer’s disease Creutzfeldt-Jakob disease Creutzfeldt-Jakob syndrome Multiinfarct dementia Dementia, vascular Huntington chorea Huntington disease Diffuse Lewy body disease Lewy body disease Parkinson disease	Cannabis Cannabi* Medical cannabis Medical marijuana Marijuana Marihuana Sativex Dronabinol Nabiximols THC Cannabis sp Epidiolex Marinol Hashish Hash oil
**MEDLINE (Ovid MEDLINE® Epub Ahead of Print, in-process and other non-indexed citations, Ovid MEDLINE ® Daily and Ovid MEDLINE ® 1946 to present)**	
Dementia-related terms	Cannabis-related terms
Parkinson disease Parkinson’s disease Dementia Dementias Alzheimer’s disease Creutzfeldt-Jakob syndrome Creutzfeldt-Jakob syndrome Dementia, vascular Dementia, vascular Huntington disease Huntington’s disease Lewy body disease	Medical marijuana Cannabis Cannabi* Marijuana Marihuana Sativex Nabiximols Dronabinol THC Cannabis sp Nabilone Epidiolex
**IPA: (International Pharmaceutical Abstracts 1970 to September 2017)**	
Dementia-related terms	Cannabis-related terms
Dementia Alzheimer’s disease Creutzfedlt-Jakob syndrome Dementia, vascular Huntington disease Lewy body disease Parkinson disease	medical marijuana cannabi* Sativex Marihuana Marijuana THC Cannabis sp Nabilone Dronabinol Epidiolex Nabiximols
**FDA: (Food and Drug Administration Clinical Trials Database, inception through August 2021)**	
Behavioral and psychological symptoms of dementia Neuropsychiatric symptoms	Cann* Cannabinoid Cannabis Marijuana

*signifies a truncation command in the search strategy permitting any term that has the text preceding the asterix

## Appendix 2

Table 3

**Table 3:** Modified downs and black checklist (based on (MacLehose et al. 2000))

Item	Criteria	Score
**Reporting**		
1	Is the hypothesis/aim/objective of the study clearly described?	Yes = 1 No = 0
2	Are the main outcomes to be measured clearly described in the Introduction or Methods section? If the primary outcomes are first mentioned in the Results section, the question should be answered no.	Yes = 1 No = 0
3	Are the characteristics of the patients included in the study clearly described? In cohort studies and trials, inclusion or exclusion criteria should be given. In case-control studies, a case-definition and the source for controls should be given.	Yes = 1 No = 0
4	Are the interventions of interest clearly described? Treatments and placebo (where relevant) that are to be compared should be clearly described.	Yes = 1 No = 0
5	Are the distributions of principal confounders in each group of subjects to be compared clearly described? A list of principal confounders is provided.	Yes = 1 No = 0
6	Are the main findings of the study clearly described? Simple outcome data (including denominators and numerators) should be reported for all significant findings so that the reader can check the major analyses and conclusions. (This question does not cover statistical tests, which are considered below).	Yes = 1 No = 0
7	Does the study provide estimates of the random variability in the data for the primary outcomes? In non-normally distributed data the inter-quartile range of results should be reported. In normally distributed data, the standard error, standard deviation or confidence intervals should be reported. If the data distribution is not described, it must be assumed that the estimates used were appropriate, and the question should be answered yes.	Yes = 1 No = 0
8	Have all significant adverse events that may be a consequence of the intervention been reported? This should be answered yes if the study demonstrates a comprehensive attempt to measure adverse events. (A list of possible adverse events is provided).	Yes = 1 No = 0
9	Have the characteristics of patients lost to follow-up been described? This should be answered yes where there were no losses to follow-up or where losses to follow-up were so small that findings would be unaffected by their inclusion. This should be answered no, where a study does not report the number of patients lost to follow-up.	Yes = 1 No = 0
10	Have actual probability values been reported (e.g. 0.035 rather than < 0.05) for the primary outcomes except where the probability value is less than 0.001?	Yes = 1 No = 0
**External validity**		
11	Were the subjects asked to participate in the study representative of the entire population from which they were recruited? The study must identify the source population for patients and describe how the patients were selected. Patients would represent the entire source population, an unselected sample of consecutive patients, or a random sample. Random sampling is only feasible where a list of all members of the relevant population exists. A study does not report the proportion of the source population from which the patients are derived; the question should be answered as unable to determine.	Yes = 1 No = 0 Unable to Determine = 0
12	Were those subjects who were prepared to participate representative of the entire population from which they were recruited? The proportion of those asked who agreed should be stated. Validation that the sample was representative would include demonstrating that the main confounding factors' distribution was the same in the study sample and the source population.	Yes = 1 No = 0 Unable to Determine = 0
13	Were the staff, places, and facilities where the patients were treated representative of most patients' treatment? For the question to be answered yes, the study should demonstrate that the intervention was representative of that in use in the source population. The question should be answered no if, for example, the intervention was undertaken in a specialist center unrepresentative of the hospitals most of the source population would attend.	Yes = 1 No = 0 Unable to Determine = 0
**Internal validity–bias**		
14	Was an attempt made to blind study subjects to the intervention they have received? For studies where the patients would have no way of knowing which intervention they received, this should be answered yes.	Yes = 1 No = 0 Unable to Determine = 0
15	Was an attempt made to blind those measuring the primary outcomes of the intervention?	Yes = 1 No = 0 Unable to Determine = 0
16	If any of the study results were based on “data dredging,” was this made clear? Any analyses that had not been planned at the outset of the study should be indicated. If no retrospective unplanned subgroup analyses were reported, then answer yes.	Yes = 1 No = 0 Unable to Determine = 0
17	In trials and cohort studies, do the analyses adjust for different lengths of patients’ follow-up, or in case-control studies, is the period between the intervention and outcome the same for cases and controls? Where follow-up was the same for all study patients, the answer should yes. If different follow-up lengths were adjusted for, for example, survival analysis, the answer should be yes. Studies where differences in follow-up are ignored, should be answered no.	Yes = 1 No = 0 Unable to Determine = 0
18	Were the statistical tests used to assess the primary outcomes appropriate? The statistical techniques used must be appropriate to the data. For example, nonparametric methods should be used for small sample sizes. Where little statistical analysis has been undertaken but no evidence of bias, the question should be answered yes. If the data distribution (normal or not) is not described, it must be assumed that the estimates used were appropriate, and the question should be answered yes.	Yes = 1 No = 0 Unable to Determine = 0
19	Was compliance with the intervention/s reliable? There was non-compliance with the allocated treatment or contamination of one group. The question should be answered no. For studies where the effect of any misclassification was likely to bias any association to the null, the question should be answered yes.	Yes = 1 No = 0 Unable to Determine = 0
20	Were the primary outcome measures used accurately (valid and reliable)? For studies where the outcome measures are clearly described, the question should be answered yes. For studies that refer to other work or demonstrate that the outcome measures are accurate, the question should be answered yes.	Yes = 1 No = 0 Unable to Determine = 0
**Internal validity–confounding (selection bias)**		
21	Were the patients in different intervention groups (trials and cohort studies), or were the cases and controls (case-control studies) recruited from the same population? For example, patients for all comparison groups should be selected from the same hospital. The question should be answered, unable to determine for cohort and case-control studies where there is no information concerning the source of patients included in the study.	Yes = 1 No = 0 Unable to Determine = 0
22	Were study subjects in different intervention groups (trials and cohort studies), or were the cases and controls (case-control studies) recruited over the same period? For a study that does not specify the period over which patients were recruited, the question should be answered as unable to determine.	Yes = 1 No = 0 Unable to Determine = 0
23	Were study subjects randomized to intervention groups? Studies that state that subjects were randomized should be answered yes except where randomization would not ensure random allocation. For example, the alternate allocation would score no because it is predictable.	Yes = 1 No = 0 Unable to Determine = 0
24	Was the randomized intervention assignment concealed from both patients and health care staff until recruitment was complete and irrevocable? All non-randomized studies should be answered no. If the assignment was concealed from patients but not from staff, it should be answered no.	Yes = 1 No = 0 Unable to Determine = 0
25	Was there an adequate adjustment for confounding in the analyses from which the main findings were drawn? This question should be answered no for trials if: the main conclusions of the study were based on analyses of treatment rather than an intention to treat; the distribution of known confounders in the different treatment groups was not described, or the distribution of known confounders differed between the treatment groups but was not taken into account in the analyses. In non-randomized studies, if the effect of the main confounders was not investigated or confounding was demonstrated. Still, no adjustment was made in the final analyses. The question should be answered as no.	Yes = 1 No = 0 Unable to Determine = 0
26	Were losses of patients to follow-up taken into account? If the numbers of patients lost to follow-up are not reported, the question should be answered as unable to determine. If the proportion lost to follow-up was too small to affect the main findings, the question should be answered yes.	Yes = 1 No = 0 Unable to Determine = 0
**Power**		
27^a^	Did the study have sufficient power to detect a clinically significant effect where the probability value for a difference is due to chance is less than 5%? Sample sizes have been calculated to detect a difference of x% and y%.	Yes 1 No 0 Unable to Determine 0

^a^Altered from Downs and Black checklist (Downs and Black 1998)

## Appendix 3

Table 4

## Appendix 4

Table 5

## References

[CR1] Abuhasira R, Schleider LB, Mechoulam R, Novack V (2018). Epidemiological characteristics, safety and efficacy of medical cannabis in the elderly. Eur J Intern Med..

[CR2] Ahmed AI, van den Elsen GA, Colbers A, Kramers C, Burger DM, van der Marck MA (2015). Safety, pharmacodynamics, and pharmacokinetics of multiple oral doses of delta-9-tetrahydrocannabinol in older persons with dementia. Psychopharmacology (Berl)..

[CR3] Alzheimer Society of Canada (2010). Rising Tide: The Impact of Dementia on Canadian Society.

[CR4] American Geriatrics Society (2015). Updated Beers Criteria for Potentially Inappropriate Medication Use in Older Adults. J Am Geriatr Soc..

[CR5] Ashton CH, Moore PB, Gallagher P, Young AH (2005). Cannabinoids in bipolar affective disorder: a review and discussion of their therapeutic potential. J Psychopharmacol..

[CR6] Bahji A, Meyyappan AC, Hawken ER (2020). Cannabinoids for the Neuropsychiatric Symptoms of Dementia: A Systematic Review and Meta-Analysis. Can J Psychiatry..

[CR7] Bahji A, Stephenson C, Tyo R, Hawken ER, Seitz DP (2020). Prevalence of cannabis withdrawal symptoms among people with regular or dependent use of cannabinoids: a systematic review and meta-analysis. JAMA Netw Open..

[CR8] Balash Y, Bar-Lev Schleider L, Korczyn AD, Shabtai H, Knaani J, Rosenberg A (2017). Medical Cannabis in Parkinson Disease: Real-Life Patients' Experience. Clin Neuropharmacol..

[CR9] Baldwin DS, Anderson IM, Nutt DJ, Bandelow B, Bond A, Davidson JR (2005). Evidence-based guidelines for the pharmacological treatment of anxiety disorders: recommendations from the British Association for Psychopharmacology. J Psychopharmacol..

[CR10] Ballard C, Waite J (2006). The effectiveness of atypical antipsychotics for the treatment of aggression and psychosis in Alzheimer's disease. Cochrane Database Syst Rev.

[CR11] Basavarajappa BS, Shivakumar M, Joshi V, Subbanna S (2017). Endocannabinoid system in neurodegenerative disorders. J Neurochem..

[CR12] Benoit M, Dygai I, Migneco O, Robert PH, Bertogliati C, Darcourt J (1999). Behavioral and psychological symptoms in Alzheimer's disease. Relation between apathy and regional cerebral perfusion. Dement Geriatr Cogn Disord..

[CR13] Bierman EJ, Comijs HC, Jonker C, Scheltens P, Beekman AT (2009). The effect of anxiety and depression on decline of memory function in Alzheimer's disease. Int Psychogeriatr..

[CR14] Bisogno T, Hanus L, De Petrocellis L, Tchilibon S, Ponde DE, Brandi I (2001). Molecular targets for cannabidiol and its synthetic analogues: effect on vanilloid VR1 receptors and on the cellular uptake and enzymatic hydrolysis of anandamide. Br J Pharmacol..

[CR15] Bruce D, Brady JP, Foster E, Shattell M (2018). Preferences for medical marijuana over prescription medications among persons living with chronic conditions: alternative, complementary, and tapering uses. J Altern Complement Med..

[CR16] Brunt TM, van Genugten M, Honer-Snoeken K, van de Velde MJ, Niesink RJ (2014). Therapeutic satisfaction and subjective effects of different strains of pharmaceutical-grade cannabis. J Clin Psychopharmacol..

[CR17] Carroll CB, Bain PG, Teare L, Liu X, Joint C, Wroath C (2004). Cannabis for dyskinesia in Parkinson disease: a randomized double-blind crossover study. Neurology..

[CR18] Caterina MJ, Julius D (2001). The vanilloid receptor: a molecular gateway to the pain pathway. Annu Rev Neurosci..

[CR19] Cerejeira J, Lagarto L, Mukaetova-Ladinska EB (2012). Behavioral and psychological symptoms of dementia. Front Neurol..

[CR20] Chagas MH, Eckeli AL, Zuardi AW, Pena-Pereira MA, Sobreira-Neto MA, Sobreira ET (2014). Cannabidiol can improve complex sleep-related behaviours associated with rapid eye movement sleep behaviour disorder in Parkinson's disease patients: a case series. J Clin Pharm Ther..

[CR21] Chagas MH, Zuardi AW, Tumas V, Pena-Pereira MA, Sobreira ET, Bergamaschi MM (2014). Effects of cannabidiol in the treatment of patients with Parkinson's disease: an exploratory double-blind trial. J Psychopharmacol..

[CR22] Cloak N, Al Khalili Y. Behavioral And Psychological Symptoms In Dementia. In: StatPearls [Internet]. Treasure Island (FL): StatPearls Publishing; 2021. 31855379

[CR23] Consroe P, Laguna J, Allender J, Snider S, Stern L, Sandyk R (1991). Controlled clinical trial of cannabidiol in Huntington's disease. Pharmacol Biochem Behav..

[CR24] Crippa JA, Derenusson GN, Ferrari TB, Wichert-Ana L, Duran FL, Martin-Santos R (2011). Neural basis of anxiolytic effects of cannabidiol (CBD) in generalized social anxiety disorder: a preliminary report. J Psychopharmacol..

[CR25] Curtis A, Mitchell I, Patel S, Ives N, Rickards H (2009). A pilot study using nabilone for symptomatic treatment in Huntington's disease. Mov Disord..

[CR26] De Deyn PP, Rabheru K, Rasmussen A, Bocksberger JP, Dautzenberg PL, Eriksson S (1999). A randomized trial of risperidone, placebo, and haloperidol for behavioral symptoms of dementia. Neurology..

[CR27] Di Marzo V, Piscitelli F (2015). The endocannabinoid system and its modulation by phytocannabinoids. Neurotherapeutics..

[CR28] Downs SH, Black N (1998). The feasibility of creating a checklist for the assessment of the methodological quality both of randomised and non-randomised studies of health care interventions. J Epidemiol Community Health..

[CR29] Fattore L, Fratta W (2010). How important are sex differences in cannabinoid action?. Br J Pharmacol..

[CR30] Ferber SG, Namdar D, Hen-Shoval D, Eger G, Koltai H, Shoval G (2020). The "Entourage Effect": terpenes coupled with cannabinoids for the treatment of mood disorders and anxiety disorders. Curr Neuropharmacol..

[CR31] Fernandez-Ruiz J, Romero J, Ramos JA (2015). Endocannabinoids and Neurodegenerative Disorders: Parkinson's Disease, Huntington's Chorea, Alzheimer's Disease, and Others. Handb Exp Pharmacol..

[CR32] Fusar-Poli P, Crippa JA, Bhattacharyya S, Borgwardt SJ, Allen P, Martin-Santos R (2009). Distinct effects of {delta}9-tetrahydrocannabinol and cannabidiol on neural activation during emotional processing. Arch Gen Psychiatry..

[CR33] Gelderblom H, Wustenberg T, McLean T, Mutze L, Fischer W, Saft C (2017). Bupropion for the treatment of apathy in Huntington's disease: A multicenter, randomised, double-blind, placebo-controlled, prospective crossover trial. PLoS One..

[CR34] Herrmann N, Ruthirakuhan M, Gallagher D, Verhoeff N, Kiss A, Black SE (2019). Randomized Placebo-Controlled Trial of Nabilone for Agitation in Alzheimer's Disease. Am J Geriatr Psychiatry..

[CR35] Ikeda M, Fukuhara R, Shigenobu K, Hokoishi K, Maki N, Nebu A (2004). Dementia associated mental and behavioural disturbances in elderly people in the community: findings from the first Nakayama study. J Neurol Neurosurg Psychiatry..

[CR36] Liberati A, Altman DG, Tetzlaff J, Mulrow C, Gotzsche PC, Ioannidis JP (2009). The PRISMA statement for reporting systematic reviews and meta-analyses of studies that evaluate health care interventions: explanation and elaboration. PLoS Med..

[CR37] Linden M, Bar T, Helmchen H (2004). Prevalence and appropriateness of psychotropic drug use in old age: results from the Berlin Aging Study (BASE). Int Psychogeriatr..

[CR38] Liu CS, Chau SA, Ruthirakuhan M, Lanctot KL, Herrmann N (2015). Cannabinoids for the treatment of agitation and aggression in Alzheimer's Disease. CNS Drugs..

[CR39] Lopez-Sendon Moreno JL, Garcia Caldentey J, Trigo Cubillo P, Ruiz Romero C, Garcia Ribas G, Alonso Arias MA (2016). A double-blind, randomized, cross-over, placebo-controlled, pilot trial with Sativex in Huntington's disease. J Neurol..

[CR40] Lotan I, Treves TA, Roditi Y, Djaldetti R (2014). Cannabis (medical marijuana) treatment for motor and non-motor symptoms of Parkinson disease: an open-label observational study. Clin Neuropharmacol..

[CR41] Lu HC, Mackie K. Review of the Endocannabinoid System. Biol Psychiatry Cogn Neurosci Neuroimaging. 2020. 10.1016/j.bpsc.2020.07.016 PMC7855189 32980261

[CR42] MacLehose RR, Reeves BC, Harvey IM, Sheldon TA, Russell IT, Black AM (2000). A systematic review of comparisons of effect sizes derived from randomised and non-randomised studies. Health Technol Assess..

[CR43] Mahlberg R, Walther S (2007). Actigraphy in agitated patients with dementia. Monitoring treatment outcomes. Z Gerontol Geriatr.

[CR44] Mahvan TD, Hilaire ML, Mann A, Brown A, Linn B, Gardner T (2017). Marijuana Use in the Elderly: Implications and Considerations. Consult Pharm..

[CR45] Marchese G, Casti P, Ruiu S, Saba P, Sanna A, Casu G (2003). Haloperidol, but not clozapine, produces dramatic catalepsy in delta9-THC-treated rats: possible clinical implications. Br J Pharmacol..

[CR46] Mesnage V, Houeto JL, Bonnet AM, Clavier I, Arnulf I, Cattelin F (2004). Neurokinin B, neurotensin, and cannabinoid receptor antagonists and Parkinson disease. Clin Neuropharmacol..

[CR47] Mintzer JE, Hoernig KS, Mirski DF (1998). Treatment of agitation in patients with dementia. Clin Geriatr Med..

[CR48] Ott A, Breteler MM, van Harskamp F, Stijnen T, Hofman A (1998). Incidence and risk of dementia. The Rotterdam Study. Am J Epidemiol.

[CR49] Public Health Agency of Canada (2014). Mapping connections: an understanding of neurological conditions in Canada: the National Population Health Study of Neurological Conditions.

[CR50] Reekes TH, Higginson CI, Ledbetter CR, Sathivadivel N, Zweig RM, Disbrow EA (2020). Sex specific cognitive differences in Parkinson disease. NPJ Parkinsons Dis..

[CR51] Reus VI, Fochtmann LJ, Eyler AE, Hilty DM, Horvitz-Lennon M, Jibson MD (2016). The American Psychiatric Association Practice Guideline on the Use of Antipsychotics to Treat Agitation or Psychosis in Patients With Dementia. Am J Psychiatry..

[CR52] Riebe CJ, Hill MN, Lee TT, Hillard CJ, Gorzalka BB (2010). Estrogenic regulation of limbic cannabinoid receptor binding. Psychoneuroendocrinology..

[CR53] Seeman P (2016). Cannabidiol is a partial agonist at dopamine D2High receptors, predicting its antipsychotic clinical dose. Transl Psychiatry..

[CR54] Shelef A, Barak Y, Berger U, Paleacu D, Tadger S, Plopsky I (2016). Safety and efficacy of medical cannabis oil for behavioral and psychological symptoms of dementia: an-open label, add-on, pilot study. J Alzheimers Dis..

[CR55] Sherman C, Ruthirakuhan M, Vieira D, Lanctot KL, Herrmann N (2018). Cannabinoids for the treatment of neuropsychiatric symptoms, pain and weight loss in dementia. Curr Opin Psychiatry..

[CR56] Shohet A, Khlebtovsky A, Roizen N, Roditi Y, Djaldetti R (2017). Effect of medical cannabis on thermal quantitative measurements of pain in patients with Parkinson's disease. Eur J Pain..

[CR57] Sieradzan KA, Fox SH, Hill M, Dick JP, Crossman AR, Brotchie JM (2001). Cannabinoids reduce levodopa-induced dyskinesia in Parkinson's disease: a pilot study. Neurology..

[CR58] Suh GH, Greenspan AJ, Choi SK (2006). Comparative efficacy of risperidone versus haloperidol on behavioural and psychological symptoms of dementia. Int J Geriatr Psychiatry..

[CR59] Sultzer DL, Davis SM, Tariot PN, Dagerman KS, Lebowitz BD, Lyketsos CG (2008). Clinical symptom responses to atypical antipsychotic medications in Alzheimer's disease: phase 1 outcomes from the CATIE-AD effectiveness trial. Am J Psychiatry..

[CR60] Tampi RR, Tampi DJ, Balachandran S, Srinivasan S (2016). Antipsychotic use in dementia: a systematic review of benefits and risks from meta-analyses. Ther Adv Chronic Dis..

[CR61] van den Elsen GA, Ahmed AI, Lammers M, Kramers C, Verkes RJ, van der Marck MA (2014). Efficacy and safety of medical cannabinoids in older subjects: a systematic review. Ageing Res Rev..

[CR62] van den Elsen GA, Ahmed AI, Verkes RJ, Kramers C, Feuth T, Rosenberg PB (2015). Tetrahydrocannabinol for neuropsychiatric symptoms in dementia: A randomized controlled trial. Neurology..

[CR63] van den Elsen GA, Tobben L, Ahmed AI, Verkes RJ, Kramers C, Marijnissen RM (2017). Effects of tetrahydrocannabinol on balance and gait in patients with dementia: A randomised controlled crossover trial. J Psychopharmacol..

[CR64] van den Elsen GAH, Ahmed AIA, Verkes RJ, Feuth T, van der Marck MA, Olde Rikkert MGM (2015). Tetrahydrocannabinol in behavioral disturbances in dementia: a crossover randomized controlled trial. Am J Geriatr Psychiatry..

[CR65] van der Hiel B, Haanen J, Stokkel MPM, Peeper DS, Jimenez CR, Beijnen JH (2017). Vemurafenib plus cobimetinib in unresectable stage IIIc or stage IV melanoma: response monitoring and resistance prediction with positron emission tomography and tumor characteristics (REPOSIT): study protocol of a phase II, open-label, multicenter study. BMC Cancer..

[CR66] van der Leeuw J, Visseren FL, Woodward M, Zoungas S, Kengne AP, van der Graaf Y (2015). Predicting the effects of blood pressure-lowering treatment on major cardiovascular events for individual patients with type 2 diabetes mellitus: results from Action in Diabetes and Vascular Disease: Preterax and Diamicron MR Controlled Evaluation. Hypertension..

[CR67] Venderova K, Ruzicka E, Vorisek V, Visnovsky P (2004). Survey on cannabis use in Parkinson's disease: subjective improvement of motor symptoms. Mov Disord..

[CR68] Vigen CL, Mack WJ, Keefe RS, Sano M, Sultzer DL, Stroup TS (2011). Cognitive effects of atypical antipsychotic medications in patients with Alzheimer's disease: outcomes from CATIE-AD. Am J Psychiatry..

[CR69] Volicer L, Stelly M, Morris J, McLaughlin J, Volicer BJ (1997). Effects of dronabinol on anorexia and disturbed behavior in patients with Alzheimer's disease. Int J Geriatr Psychiatry..

[CR70] Wakley AA, Craft RM (2011). Antinociception and sedation following intracerebroventricular administration of Delta(9)-tetrahydrocannabinol in female vs. male rats. Behav Brain Res..

[CR71] Walther S, Mahlberg R, Eichmann U, Kunz D (2006). Delta-9-tetrahydrocannabinol for nighttime agitation in severe dementia. Psychopharmacology (Berl)..

[CR72] Walther S, Schupbach B, Seifritz E, Homan P, Strik W (2011). Randomized, controlled crossover trial of dronabinol, 2.5 mg, for agitation in 2 patients with dementia. J Clin Psychopharmacol..

[CR73] Wei Z, Mousseau DD, Dai Y, Cao X, Li XM (2006). Haloperidol induces apoptosis via the sigma2 receptor system and Bcl-XS. Pharmacogenomics J..

[CR74] Woodward MR, Harper DG, Stolyar A, Forester BP, Ellison JM (2014). Dronabinol for the treatment of agitation and aggressive behavior in acutely hospitalized severely demented patients with noncognitive behavioral symptoms. Am J Geriatr Psychiatry..

[CR75] Zuardi AW, Crippa JA, Hallak JE, Pinto JP, Chagas MH, Rodrigues GG (2009). Cannabidiol for the treatment of psychosis in Parkinson's disease. J Psychopharmacol..

[CR76] Zuardi AW, Shirakawa I, Finkelfarb E, Karniol IG (1982). Action of cannabidiol on the anxiety and other effects produced by delta 9-THC in normal subjects. Psychopharmacology (Berl)..

